# Quantitative pupillometry as a marker of autonomic dysregulation in vestibular migraine

**DOI:** 10.22514/jofph.2026.053

**Published:** 2026-07-12

**Authors:** Gokce Saygi Uysal, Zulkuf Kucuktag, Aykut Ozdogan, Mevlut Yilmaz, Tuba Kuz Teksut, Gulce Kirazli

**Affiliations:** ^1^Department of Otorhinolaryngology, Ankara Etlik City Hospital, 06170 Ankara, Türkiye; ^2^Department of Ophthalmology, Ankara Etlik City Hospital, 06170 Ankara, Türkiye; ^3^Department of Neurology, Ankara Etlik City Hospital, 06170 Ankara, Türkiye; ^4^Department of Audiology, Faculty of Health Sciences, Ege University, 35100 Izmir, Türkiye

**Keywords:** Vestibular migraine, Quantitative pupillometry, Autonomic function, Pupillary light reflex

## Abstract

**Background**: Vestibular migraine (VM) has been associated with altered central sensory processing; however, objective measures of autonomic involvement remain insufficiently characterized. Quantitative pupillometry enables standardized assessment of both static and dynamic pupil responses. This study compared pupillary parameters between patients with VM and healthy controls under controlled illumination conditions. **Methods**: Seventy participants were enrolled, including 40 patients diagnosed with VM according to the International Classification of Headache Disorders, 3rd edition (ICHD-3) criteria, and 30 age- and sex-matched controls. Bilateral pupillometry was performed using an automated infrared system under scotopic, mesopic, low photopic, high photopic, and resting light conditions. Static pupil diameter and dynamic parameters (including constriction amplitude, latency, duration, velocity, and dilation metrics) were recorded. Between-group comparisons were conducted using parametric or nonparametric tests, as appropriate based on the data distribution. **Results**: No significant differences were observed between groups in mean pupil diameter across illumination conditions (all *p* > 0.05). Dynamic pupillary parameters also did not differ significantly between the study groups. However, both groups demonstrated mild right-to-left asymmetry in static measurements, with a statistically significant interocular difference observed in the VM group under high-photopic conditions (*p* = 0.001). Dynamic asymmetries were minimal and not clinically meaningful. **Conclusions**: Interictal static and dynamic pupillary parameters did not differ significantly between patients with VM and healthy controls. Although minor light-dependent asymmetries were observed, their clinical significance remains uncertain. Further studies incorporating longitudinal designs and multimodal autonomic assessments are warranted to clarify the relationship between autonomic function and VM.

## 1. Introduction

Migraine is one of the most prevalent and disabling primary headache disorders, affecting approximately 13–15% of the global population and exhibiting a marked female predominance, with a female-to-male ratio of nearly 3:1 [1, 2, 3]. Despite its high prevalence, migraine remains substantially underdiagnosed and undertreated, imposing a considerable burden on both patients and healthcare systems. Vestibular symptoms are reported in nearly half of individuals with migraine, further highlighting the close neurobiological interaction between migraine mechanisms and vestibular pathways [4].

Vestibular migraine (VM) is a commonly underdiagnosed condition encountered in general medical, headache, and neuro-otology clinics. It is recognized as a distinct clinical entity that reflects the convergence of migraine-related and vestibular mechanisms, as defined in the International Classification of Headache Disorders, 3rd edition (ICHD-3) [5]. Clinically, VM typically presents with recurrent episodes of vertigo or motion intolerance accompanied by migrainous features, such as headache, photophobia, or phonophobia. According to the diagnostic criteria jointly proposed by the International Headache Society and the Bárány Society in 2012, the diagnosis requires at least five episodes of moderate to severe vestibular symptoms lasting from 5 minutes to 72 hours, a current or previous history of migraine, and the presence of migraine features, including headache, photophobia and/or phonophobia, or visual aura, during at least 50% of these episodes [5].

VM is a multifactorial disorder characterized predominantly by central sensory dysregulation and dysfunction of neuronal networks involving both cortical and brainstem pathways. Its pathophysiology is thought to involve several interacting mechanisms, including ion channel dysfunction, cortical hyperexcitability, activation of trigeminal networks, and context-dependent modulation of autonomic function [6]. Although vascular mechanisms may play a secondary modulatory influence, they are not currently regarded as the primary drivers of VM pathophysiology.

Certain genes implicated in rare monogenic migraine syndromes, including Calcium Voltage-Gated Channel Subunit Alpha1 A (*CACNA1A*), ATPase Na^+^/K^+^ Transporting Subunit Alpha 2 (*ATP1A2*), Notch Receptor 3 (*NOTCH3*), Three Prime Repair Exonuclease 1 (*TREX1*), and Collagen Type IV Alpha 1 Chain (*COL4A1*), have been associated with disorders characterized by episodic neurological dysfunction and may suggest potential shared neurogenetic pathways with familial hemiplegic migraine and epilepsy; however, these observations are derived primarily from rare hereditary conditions and therefore do not provide direct evidence for a causal role in typical VM [7, 8, 9, 10].

Although similarities between migraine and epilepsy have been proposed, particularly in relation to neuronal hyperexcitability and ion channel dysfunction, much of the available evidence comes from rare monogenic channelopathies, *i.e.*, familial hemiplegic migraine and related genetic syndromes, rather than from the more common forms seen in routine clinical practice. Therefore, it remains unclear to what extent these mechanisms are relevant to the broader population of patients with migraine [11].

Vestibular stimulation engages both cortical and subcortical regions involved in sensory integration and pain processing, including the insula, orbitofrontal cortex, and cingulate gyrus, thereby facilitating functional interactions between vestibular and nociceptive networks. Neuroimaging studies have demonstrated overlapping functional connectivity among vestibular, thalamic, and limbic regions, suggesting shared cortical substrates for sensory integration that further support the concept of central sensory dysregulation [12, 13].

Activation of trigeminal afferents and the release of vasoactive neuropeptides, such as calcitonin gene-related peptide (CGRP) and Substance P, can induce vasodilation, neurogenic inflammation, and transient ischemia, which may contribute to vertigo and auditory disturbances [14].

Cortical spreading depression (CSD) has long been proposed as a key mechanism underlying migraine with aura and may also affect visual and sensory processing [15, 16]. It is widely regarded as the electrophysiological basis of migraine with aura and is characterized by a propagating wave of neuronal and glial depolarization followed by transient suppression of cortical activity [17, 18]. However, although CSD occupies an important place in current models of migraine pathophysiology, its direct role in VM has not yet been clearly established.

The preventive effects of antiepileptic agents in VM are more likely related to their ability to modulate neuronal hyperexcitability, ion channel function, and synaptic transmission than to any specific inhibitory action on CSD [15, 16]. Current models of migraine pathophysiology place greater emphasis on dysfunction within distributed neural networks, altered sensory processing, and thalamocortical dysrhythmia. Accordingly, the clinical benefit of antiepileptic medications in VM is more likely to reflect stabilization of aberrant neuronal firing patterns rather than a direct anti-CSD mechanism.

Autonomic nervous system (ANS) dysfunction, characterized by sympathetic overactivity and reduced parasympathetic tone, may contribute to systemic manifestations such as nausea, diaphoresis, and cardiovascular alterations [19]. In addition, increased levels of inflammatory cytokines (*e.g.*, tumor necrosis factor alpha (TNF-α) and interleukin-6 (IL-6), IL-1, IL-10) have been reported, further supporting the presence of neuroinflammatory activity that may amplify these effects [20].

Elevated levels of pro-inflammatory cytokines, particularly TNF-α and IL-6, have been reported in migraine cohorts, supporting the concept of activity-dependent neuroinflammatory modulation rather than a primary inflammatory disorder [21, 22]. Within the trigeminovascular system, these neuroimmune interactions may further influence neuronal excitability and contribute to central sensitization [23].

In contrast, IL-10 is a well-recognized anti-inflammatory cytokine with important immunoregulatory functions [24]. Therefore, elevated IL-10 levels are more appropriately interpreted as a compensatory response aimed at counterbalancing pro-inflammatory signaling, rather than as evidence of exacerbated inflammatory activity [24].

Although these inflammatory mediators may influence synaptic transmission, glial activation, and the amplification of sensory network activity, current evidence does not support a distinct inflammatory biomarker profile specific to VM. Accordingly, they are more appropriately regarded as modulators of neuronal network excitability, rather than as primary drivers of disease pathogenesis.

Functional imaging and electrophysiological studies have demonstrated cortical hyperexcitability and altered sensory integration within the vestibulo-thalamo-cortical network, thereby supporting a central mechanism in VM. Although no specific biomarker has yet been established for VM, vestibular and oculomotor abnormalities, including impaired smooth pursuit, abnormal saccades, and central positional nystagmus, are frequently observed. These abnormalities may fluctuate between the ictal and interictal phases and are considered to reflect underlying central sensory dysregulation [25].

The thalamus is a key component of the trigemino-thalamo-cortical circuit, serving not only as a relay center for pain transmission, but also as an important component of the vestibular system. It receives peripheral vestibular input from the vestibular nuclei and transmits this information to the central vestibular cortex [26]. Neuroimaging studies also support the important role of the thalamus in migraine pathophysiology, with both neuroimaging and electrophysiological findings further confirming abnormalities involving vestibular, thalamic, and cortical networks [26, 27].

Given the close interaction among vestibular, trigeminal, and autonomic networks, autonomic imbalance may contribute to symptom generation in VM. Pupillometry, which quantitatively measures pupil size and its dynamic responses to light or cognitive stimuli, provides an objective and noninvasive indicator of autonomic and central nervous system function. Because pupillary behavior reflects the balance between sympathetic and parasympathetic activity, pupillometry may serve as a useful noninvasive tool for assessing autonomic regulation [28].

Accordingly, this study aimed to determine whether patients with VM exhibit alterations in pupil diameter and dynamic pupillary responses under different illumination conditions compared with healthy controls. We hypothesized that measurable changes in pupillary parameters might reflect altered autonomic modulation associated with VM.

## 2. Materials and methods

### 2.1 Participants

The study cohort initially comprised 74 participants, among whom 43 patients were diagnosed with VM according to the ICHD-3 criteria [5], and 31 healthy controls without a history of migraine or vestibular disorders. Three participants in the study group and one in the control group were excluded because of inadequate cooperation during vestibular testing. Thus, the final analysis consisted of 70 participants, comprising 40 patients with VM and 30 healthy controls.

The VM cohort consisted of patients with episodic VM diagnosed according to the ICHD-3 criteria. Among these patients, 4 (10.0%) had migraine with aura, whereas 36 (90.0%) had migraine without aura. The attack frequency ranged from 4 to 6 episodes per month, with a mean ± standard deviation (SD) of 4.9 ± 0.7 episodes per month. None of the participants met the diagnostic criteria for chronic migraine. These clinical characteristics were considered when interpreting interictal pupillometric responses.

The exclusion criteria included ocular disease, neurological or metabolic disorders, use of medications that could affect pupillary or autonomic function, and abnormal vestibular test results. All participants underwent a comprehensive otoneurological examination to exclude alternative causes of vertigo.

This study was approved by the Bakircay University Non-Interventional Clinical Research Ethics Committee (Approval No. 2116), and written informed consent was obtained from all participants at the time of hospitalization.

### 2.2 Inclusion criteria

Participants were eligible for inclusion if they met the following criteria: (1) age between 18 and 65 years; (2) normal ophthalmologic findings, including best-corrected visual acuity and normal anterior and posterior segment examinations; (3) a normal otoneurological evaluation, with no evidence of peripheral or central vestibular pathology other than VM; (4) for the VM group, fulfillment of the ICHD-3, diagnostic criteria for VM; (5) for the control group, no history of migraine, vertigo, or vestibular disorders; (6) no use of medications that could affect autonomic, neurological, or pupillary responses; and (7) provided written informed consent and could complete the full pupillometric protocol. Participant recruitment and data collection were conducted between October 2025 and November 2025.

All patients with VM were evaluated during the interictal phase, which was defined as the absence of migraine or vestibular symptoms for at least 72 hours before testing. This criterion was applied to minimize the influence of acute autonomic changes during attacks on pupillometric measurements. Nevertheless, although participants were assessed during an interictal period defined in this way, it cannot be completely excluded that some individuals were evaluated during the premonitory phase of migraine, which may precede headache onset by up to 48 hours [29].

Although the inclusion criteria allowed participants aged 18–65 years, the final sample included individuals within a narrower age range.

### 2.3 Ophthalmic examination

All participants underwent a comprehensive ophthalmic examination, which included best-corrected visual acuity assessment, slit-lamp biomicroscopy, and evaluation of both the anterior and posterior segments.

### 2.4 Pupillometric procedure

Non-contact pupillometric measurements were obtained using an automated quantitative pupillometry system (MonPack One, Metrovision, Pérenchies, France), and all examinations were performed by the same experienced clinician (Fig. 1).

**Fig. 1.** Representative pupillometry recordings obtained under different illumination conditions. (A) scotopic, (B) mesopic, (C) low photopic, and (D) high photopic illumination. (E) Representative pupillary light reflex response curve. BI: Binocular; OD: Oculus Dexter; OS: Oculus Sinister.

Bilateral pupillary measurements were recorded using an infrared pupillometer under five standardized illumination conditions: scotopic, mesopic, low photopic, high photopic, and resting light. Before testing, all subjects underwent dark adaptation in order to standardize baseline pupil diameter.

For each condition, dynamic pupillary parameters were recorded, including constriction amplitude, latency, duration, and velocity, as well as dilation latency, duration, and velocity. For statistical analysis, measurements obtained from both eyes were averaged.

The five illumination conditions were defined as follows: scotopic testing was performed under complete darkness to assess pupil responses when rod photoreceptors are predominantly active; mesopic testing evaluated pupil behavior under intermediate lighting conditions, in which both rod and cone photoreceptors contribute; low-photopic testing measured pupil responses under low-light conditions dominated mainly by cone activity; high-photopic testing examined pupillary constriction under bright illumination, when cone photoreceptors are fully activated; and resting light conditions were used to determine the natural baseline pupil diameter in the absence of a direct light stimulus.

### 2.5 Statistical analysis

The statistical analysis of the data was performed using the Statistical Package for the Social Sciences (SPSS) version 27 (IBM Corp., Armonk, NY, USA). Descriptive statistics were expressed as frequency, percentage, mean, SD, median, and minimum–maximum values. The relationships between categorical variables were analyzed using the Pearson chi-square test.

The normality of continuous variables was assessed using the Shapiro-Wilk test. Skewness and kurtosis values were also examined, and data distribution was visually evaluated using graphical methods. According to Tabachnick and Fidell, skewness and kurtosis coefficients between −1.5 and +1.5 are considered acceptable for normal distribution [30]. In the present study, all variables met this criterion.

For comparisons between groups, the independent-samples *t*-test was used for normally distributed variables, whereas the Mann-Whitney U test was used for variables that were not normally distributed. Within-group comparisons were performed using the paired-samples *t*-test or the Wilcoxon signed-rank test, as appropriate. A *p*-value of < 0.05 was considered statistically significant in all analyses.

The required sample size for the study was determined using G*Power (version 3.1.9.7, G*Power, Heinrich Heine University Düsseldorf, Düsseldorf, NRW, Germany). The effect size was estimated from previously published reference studies and calculated as Cohen’s *d* = 0.86. According to the power analysis, with a significance level of α = 0.05, a statistical power of 90% (1 − β), and an effect size of *d* = 0.86, a minimum of 30 participants per group was required. Therefore, the study was planned with a minimum sample size of 60 participants.

In addition to *p*-values, effect sizes were calculated to quantify the magnitude of the observed differences. Cohen’s *d* was used for comparisons between independent groups. Effect sizes were interpreted according to the thresholds recommended for pain research, defined as small (0.10), medium (0.30), and large (0.70) [31].

## 3. Results

In the present study, 85% of the patients and 67% of the control participants were female. There was no significant difference in sex distribution between the two groups (*p* = 0.071). The ages of the patients ranged from 18 to 58 years (mean ± SD: 37.3 ± 13.66 years), whereas those of the control group ranged from 23 to 47 years (mean ± SD: 34.33 ± 8.90 years). No significant difference in age was observed between the groups (*p* = 0.521). Thus, the patient and control groups were comparable in terms of age and sex (Table 1).

**Table 1.** Comparison of demographic characteristics between groups.

		Group		*p*
		Patient (n = 40)	Control (n = 30)	
Gender, n (%)				
	Female	34.00 (85.00)	20.00 (66.70)	0.071*
	Male	6.00 (15.00)	10.00 (33.30)	
Age in years, Mean ± SD (min–max)		37.30 ± 13.60 (16–58)	34.30 ± 8.90 (23–47)	0.521**

*: Pearson chi-square test; **: Independent samples t-test. SD: Standard Deviation; min: Minimum; max: Maximum.

Across all illumination conditions, including scotopic, mesopic, low-photopic, high-photopic, and resting light conditions, no significant differences in mean pupil diameter were observed between the VM and control groups (all *p* > 0.05; Effect Size (ES) range = 0.0–0.3, indicating predominantly small effect sizes). However, significant right-to-left asymmetry in pupil diameter was consistently observed within both groups, particularly under low-light conditions, namely the scotopic and mesopic settings. Under high-photopic conditions, a significant right-to-left difference was observed in the patient group (*p* = 0.001), whereas no such difference was found in the control group (*p* = 0.125) (Table 2).

**Table 2.** Comparison of pupil diameter values between groups.

Side		Patient		Control		*p*	ES (Cohen’s *d*)
		Mean ± SD	M (Min–Max)	Mean ± SD	M (Min–Max)		
Scotopic							
	Right	6.90 ± 0.80	7.00 (5.30–9.00)	6.90 ± 0.90	7.10 (5.00–8.50)	0.721*^t^*	Right
	Left	7.20 ± 0.80	7.20 (5.40–9.20)	7.10 ± 1.10	7.30 (5.00–9.20)		Left
	*p*	0.001*^p^*		0.001*^p^*			
Mesopic							
	Right	5.50 ± 1.10	5.50 (3.4–8.2)	5.70 ± 1.00	5.5 (3.9–7.7)	0.685*^t^*	Right
	Left	5.70 ± 1.10	5.70 (3.5–8.3)	5.80 ± 1.20	5.6 (3.7–8.6)		Left
	*p*	0.003*^p^*		0.028*^p^*			
Photopic low light							
	Right	4.20 ± 0.80	4.10 (2.9–6.3)	4.30 ± 0.70	4.20 (3.30–5.50)	0.962^m^	Right
	Left	4.40 ± 0.80	4.30 (2.9–6.1)	4.30 ± 0.70	4.40 (3.30–5.50)		Left
	*p*	0.001^w^		0.017^w^			
Photopic high light							
	Right	3.00 ± 0.30	3.00 (2.20–3.70)	3.10 ± 0.30	3.10 (2.70–3.60)	0.420*^t^*	Right
	Left	3.10 ± 0.30	3.10 (2.4–3.6)	3.10 ± 0.30	3.10 (2.60–3.70)		Left
	*p*	0.001*^p^*		0.125*^p^*			
Resting							
	Right	5.65 ± 0.75	5.68 (3.72–7.30)	5.59 ± 0.69	5.57 (4.58–6.78)	0.425*^t^*	Right
	Left	5.85 ± 0.79	5.84 (3.87–7.44)	5.7 ± 0.83	5.53 (4.32–7.12)		Left
	*p*	0.001*^p^*		0.003*^p^*			

*^p^: p-value; ^t^: independent samples t-test; ^m^: Mann-Whitney U test; ^w^: Wilcoxon signed-rank test; SD: standard deviation; M: median; Min: minimum; Max: maximum; ES: effect size (Cohen’s d).*

Across all dynamic pupillometric parameters, including contraction amplitude, latency, duration, and velocity, as well as dilation latency, duration, and velocity, no significant differences were found between the VM and control groups (all *p* > 0.05; ES range = 0.03–0.41, indicating small to moderate effect sizes) (Table 3). Minor right-to-left asymmetries in contraction velocity were detected in both groups; however, these variations were inconsistent and, given the predominantly small effect sizes, are unlikely to represent clinically meaningful differences between patients with VM and controls.

**Table 3.** Comparison of constriction and dilation values between groups.

Side		Patient		Control		*p*	ES (Cohen’s *d*)
		Mean ± SD	M (Min–Max)	Mean ± SD	ES (Cohen’s *d*)		
Constriction amplitude (mm)							
	Right	1.84 ± 0.27	1.84 (1.2–2.48)	1.90 ± 0.25	ES (Cohen’s *d*)	0.528*^t^*	Right
	Left	1.88 ± 0.29	1.88 (1.28–2.48)	1.92 ± 0.3	ES (Cohen’s *d*)		Left
	*p*	0.071*^p^*		0.159*^p^*			
Constriction latency (ms)							
	Right	241 ± 23	243 (147–282)	240 ± 35	ES (Cohen’s *d*)	0.491^m^	Right
	Left	238 ± 36	245 (110–285)	239 ± 35	ES (Cohen’s *d*)		Left
	*p*	0.528^w^		0.845^w^			
Constriction duration (ms) light							
	Right	590 ± 54	591 (457–732)	579 ± 104	ES (Cohen’s *d*)	0.188^m^	Right
	Left	580 ± 60	575 (485–721)	599 ± 65	ES (Cohen’s *d*)		Left
	*p*	0.307^w^		0.926^w^			
Constriction velocity (mm/s)							
	Right	5.86 ± 0.76	5.88 (4.24–7.5)	5.96 ± 0.90	ES (Cohen’s *d*)	0.583*^t^*	Right
	Left	6.03 ± 0.73	6.05 (4.14–7.33)	6.14 ± 0.99	ES (Cohen’s *d*)		Left
	*p*	0.029*^p^*		0.033*^p^*			
Dilation latency (msn)							
	Right	818 ± 102	832 (298–975)	845 ± 62	ES (Cohen’s *d*)	0.548*^t^*	Right
	Left	807 ± 100	802 (299–956)	840 ± 49	ES (Cohen’s *d*)		Left
	*p*	0.213*^p^*		0.519*^p^*			
Dilation duration (ms)							
	Right	1642 ± 71	1637 (1359–1762)	1633 ± 77	ES (Cohen’s *d*)	0.064^m^	Right
	Left	1651 ± 73	1665 (1455–1791)	1641 ± 69	ES (Cohen’s *d*)		Left
	*p*	0.762^w^		0.184^w^			
Dilation velocity (mm/s)							
	Right	1.73 ± 0.28	1.71 (1.15–2.30)	1.77 ± 0.4	ES (Cohen’s *d*)	0.674*^t^*	Right
	Left	1.74 ± 0.31	1.74 (1.1–2.45)	1.78 ± 0.36	ES (Cohen’s *d*)		Left
	*p*	0.746*^p^*		0.504*^p^*			

*^p^: p-value; ^t^: independent samples t-test; ^m^: Mann-Whitney U test; ^w^: Wilcoxon signed-rank test; SD: standard deviation; M: median; Min: minimum; Max: maximum; ES: effect size (Cohen’s d).*

## 4. Discussion

### 4.1 Pupillometric and autonomic findings

In the present study, no significant interictal differences in pupillometric parameters were observed between patients with VM and healthy controls. However, autonomic dysfunction in migraine is known to be variable and phase-dependent [32, 33], and interictal pupillometry may have limited sensitivity for detecting subtle central alterations. Consistent with this interpretation, the observed differences were associated with predominantly small effect sizes, suggesting that they are unlikely to reflect clinically meaningful autonomic alterations in VM during the interictal phase.

The photopic pupillary asymmetries observed in some participants should, therefore, be interpreted with caution. Although mild anisocoria may fall within the range of physiological variability [34], persistent asymmetries may still suggest the possibility of asymmetric central autonomic modulation. Nevertheless, pupillometry does not allow localization of neural activity to specific brainstem nuclei [35], and therefore, our findings cannot be attributed to lateralized activity in structures such as the locus coeruleus or the periaqueductal gray.

Taken together, these findings suggest that interictal pupillometry provides limited, but potentially complementary, information regarding autonomic regulation in VM. Its role as a standalone biomarker remains uncertain. Future studies integrating multimodal autonomic assessments with functional neuroimaging may help determine whether these subtle pupillary variations reflect central network-level modulation rather than peripheral influences or physiological variability.

### 4.2 Light-dependent modulation and brainstem mechanisms

In bright light, patients with VM exhibited a noticeable right-to-left asymmetry in pupil responses. In a similar context, Zou *et al*. [36] reported that patients with VM showed increased photophobia and greater sensitivity to visual triggers, both of which were significantly associated with more severe vertigo symptoms.

Their study further demonstrated that photophobia during VM attacks was positively correlated with interictal photosensitivity and visually triggered dizziness, thereby highlighting the contribution of visual hypersensitivity to VM and supporting the need for individualized management and preventive strategies [36].

This pattern of modulation likely reflects transient, centrally mediated hypersensitivity within the Locus Coeruleus–Periaqueductal Gray Mater (LC–PAG) network rather than fixed structural autonomic dysfunction. The increased reactivity observed under bright light may indicate that VM is associated with a functional, stimulus-dependent imbalance in sympathetic–parasympathetic integration, which is consistent with the broader concept of central sensory hypersensitivity in migraine. In addition, pupil dynamics may serve as an indirect marker of LC-mediated coordination between cognitive and autonomic activity across distributed neural networks [37].

### 4.3 Comparison with previous studies and broader interpretation

While previous studies have reported subtle interictal autonomic or pupillary asymmetries in certain migraine subtypes, our findings indicate only minimal interictal differences in patients with VM, suggesting that pupillometric changes may be episodic, stimulus-dependent, or subtype-specific rather than consistently detectable across all migraine phenotypes.

Kavuncu *et al*. [38] reported significant inter-eye differences in scotopic pupil diameter, together with shortened constriction latency in patients with migraine with aura, suggesting a shift in pupillary balance toward parasympathetic predominance.

Similarly, Harle *et al*. [39] demonstrated interictal interocular differences in pupillary latency in patients with migraine, indicating that subtle autonomic asymmetry may persist even between attacks.

Against this background, our findings suggest that the light-dependent and lateralized pupillary asymmetry observed in VM may reflect a residual but adaptive form of central autonomic modulation rather than fixed structural impairment. This interpretation is consistent with the framework of LC–PAG-mediated hypersensitivity within brainstem autonomic circuits and further supports the concept of brainstem-centered functional dysregulation in VM. These findings suggest that VM is not characterized by fixed or degenerative autonomic deficits, but rather by reversible and context-dependent modulation of central autonomic control. The subtle and light-dependent asymmetry identified in the present study may therefore represent a physiological expression of the brainstem’s dynamic regulation of sensory and autonomic processes. In this context, pupillometry may serve as a useful and noninvasive approach for detecting phase-dependent autonomic alterations and for exploring brainstem network involvement in VM.

Together, these pupillometric findings further highlight the possible contribution of brainstem autonomic nuclei and their thalamocortical connections to the pathophysiology of VM. To better interpret these results, it is important to consider how autonomic dysregulation interacts with vestibular, trigeminal, and cortical circuits in producing the complex clinical manifestations of VM.

### 4.4 Autonomic dysregulation in vestibular migraine

The ANS maintains physiological homeostasis through a dynamic balance between sympathetic and parasympathetic activity, and disruption of this equilibrium in migraine may result in sympathetic hyperactivity or parasympathetic hypoactivity, particularly during attacks [40].

Autonomic regulation within interconnected vestibular–trigeminal–brainstem networks represents an important modulatory component of VM pathophysiology [41, 42]. While the ANS contributes to clinical manifestations such as nausea, vertigo, and light sensitivity, current evidence suggests that these changes reflect phase-dependent modulation of central sensory networks rather than primary structural autonomic dysfunction [40].

Functional imaging studies have demonstrated altered connectivity among the hypothalamus, thalamus, brainstem nuclei, and vestibular cortex, regions that collectively mediate sensory and autonomic integration. This network-level dysregulation likely contributes to symptoms such as dizziness, nausea, cardiovascular instability, and photophobia commonly observed in VM [40].

At the brainstem level, reciprocal connections among the vestibular nuclei, nucleus tractus solitarius, dorsal motor nucleus of the vagus, and locus coeruleus form a vestibulo-autonomic network that enables bidirectional modulation between vestibular input and autonomic output. Activation of the trigeminovascular system, together with the release of vasoactive neuropeptides such as calcitonin gene-related peptide (CGRP) and substance P, induces neurogenic inflammation and vasodilation [14], which may transiently influence vestibulo-ocular reflex control and autonomic tone, thereby contributing to clinical symptoms such as vertigo, motion intolerance, and lightheadedness. Beyond these autonomic circuits, the vestibular nuclei themselves may undergo sensitization under the influence of migraine-related brainstem regions, with additional modulation provided by inhibitory cerebellar feedback from the nodulus and uvula, where canal–otolith integration occurs [43].

Although CGRP and substance P are well-established mediators in migraine pathophysiology, their direct effects on vestibulo-ocular reflex (VOR) control have not been experimentally established. CGRP primarily modulates nociceptive transmission and vascular tone [22]. Accordingly, our findings do not support a direct mechanistic link between these neuropeptides and VOR disruption.

The thalamus acts as a central relay that integrates vestibular and nociceptive inputs. Its higher-order nuclei, including the posterior, lateral posterior, and lateral dorsal nuclei, project to cortical regions involved in multisensory processing [26, 44]. Functional imaging studies have demonstrated increased thalamic activation during both vestibular stimulation and migraine episodes [26, 45], supporting the concept of dynamic, phase-dependent central sensory dysregulation rather than fixed structural sensitization.

At the cortical level, patients with migraine exhibit increased excitability, which may be influenced by modulatory input from the locus coeruleus and dorsal raphe nuclei [46, 47]. In addition, abnormal cortical interactions between visual and vestibular networks have been demonstrated in VM, contributing to heightened motion sensitivity and increased susceptibility to motion sickness, even during interictal periods [48, 49].

Collectively, these findings indicate that VM reflects multilevel central dysregulation involving brainstem autonomic nuclei, thalamic relay structures, and cortical sensory networks. Taken together, these mechanisms support the interpretation of VM as a functional and dynamic disorder characterized by transient hypersensitivity within the vestibulo-autonomic axis rather than structural damage. This integrated framework also links the pupillometric evidence of brainstem asymmetry with broader processes of central sensitization across vestibular and nociceptive networks.

### 4.5 Comparison with previous research and physiological interpretation

Earlier studies in migraine without vestibular involvement have demonstrated reduced baseline pupil diameter and shortened constriction latency, reflecting an accelerated pupillary light response, findings that have been interpreted as reflecting relative parasympathetic predominance [38].

In contrast, our results are consistent with those of Gufoni and Casani [50], who reported no significant interictal pupillary asymmetry, suggesting that autonomic dysfunction in VM may be episodic and phase-dependent. The stronger intragroup correlations observed in VM patients may further indicate central reorganization within the vestibulo-autonomic network rather than peripheral impairment, implying that pupillary changes may represent adaptive responses to fluctuating cortical excitability.

Previous pupillometric studies in migraine have yielded heterogeneous findings depending on the subtype and timing of assessment. Harle *et al*. [39] demonstrated increased interocular differences in pupillary light reflex latency and a mild imbalance between sympathetic and parasympathetic activity that persisted during the interictal phase, suggesting subtle autonomic asymmetry even outside headache periods. Kavuncu *et al*. [38], in contrast, reported a shift in pupillary balance toward parasympathetic predominance in patients with migraine with aura, reflected by prolonged constriction latency and reduced constriction velocity.

In our present study, no overall interictal differences were observed in static or dynamic pupillary parameters between patients with VM and healthy controls; however, a significant right-to-left asymmetry was detected under high-photopic illumination. This pattern differs from the more generalized parasympathetic predominance described in migraine with aura and instead suggests a stimulus-dependent, lateralized modulation of central autonomic pathways, particularly within midbrain networks, such as the locus coeruleus and periaqueductal gray, which are involved in light sensitivity and pain processing.

Taken together, these findings suggest that migraine-related autonomic dysfunction is functional and context-dependent rather than structural, with distinct patterns across migraine subtypes. Whereas migraine with aura may be associated with a more sustained parasympathetic bias, VM appears to involve dynamic, light-dependent asymmetry consistent with transient, centrally mediated hypersensitivity within the vestibulo-autonomic network. Compared with previous clinical studies that assessed pupil diameter under limited conditions, the present study evaluated both static and dynamic pupillary responses across multiple illumination levels, which represents a methodological strength and allows for a more comprehensive characterization of autonomic function and light-dependent modulation in VM.

Our present study also investigated interictal pupillary dynamics in patients with VM and compared them with those of healthy controls. The absence of significant differences in baseline pupillometric measures suggests that interictal autonomic regulation may appear largely preserved; however, autonomic dysfunction in migraine is known to be variable, phase-dependent, and non-linear, and interictal pupillometry may, therefore, have limited sensitivity for detecting subtle central disturbances [32, 33]. Accordingly, the absence of statistically significant differences should not be interpreted as definitive evidence of normal autonomic function in all patients with VM.

Interictal pupillometry may have limited sensitivity for detecting subtle disturbances of central autonomic regulation. Although minor pupillary asymmetries were observed, these findings may reflect physiological variability rather than true pathological alterations. At present, the clinical utility of pupillometry as a diagnostic tool or early biomarker in VM remains uncertain, particularly because data on its sensitivity, specificity, and reproducibility in clinical settings are still lacking. Therefore, it would be premature to regard pupillometry as a reliable method for identifying early markers of the disorder. Future studies incorporating larger sample sizes, standardized protocols, and multimodal autonomic and neurophysiological assessments are needed to clarify its potential value and to further elucidate the complex network-level mechanisms underlying VM. Collectively, these findings support the concept that VM is primarily a disorder of dynamic network dysfunction rather than fixed structural autonomic impairment.

### 4.6 Clinical implications

From a clinical perspective, these findings suggest that pupillometry may serve as a non-invasive and objective tool for evaluating autonomic function in VM. Although no persistent interictal abnormalities were identified, the subtle, light-dependent asymmetries observed in patients with VM may indicate context-related alterations in central autonomic regulation. Assessment of pupillary responses under different illumination conditions may, therefore, contribute to a better understanding of the autonomic mechanisms associated with vestibular and migraine symptoms. Future studies should incorporate ictal-phase evaluations, heart rate variability, and functional neuroimaging in order to clarify the temporal dynamics of autonomic activity in VM and to further assess the potential diagnostic and prognostic relevance of pupillometry.

## 5. Conclusions

In conclusion, VM appears to involve dynamic and reversible modulation of central autonomic control rather than persistent structural impairment. Pupillary behavior in VM may reflect this functional interplay, and the light-dependent asymmetries observed in this study may indicate subtle lateralized hypersensitivity within central autonomic pathways. However, the predominantly small effect sizes further support the absence of clinically meaningful autonomic alterations during the interictal phase. Consistent with this, interictal pupillometric parameters did not differ significantly between patients with VM and healthy controls, suggesting that baseline autonomic regulation remains largely preserved under standardized conditions. Although minor numerical differences were observed in some pupillometric parameters, the corresponding effect sizes were small, indicating that these variations are unlikely to represent clinically meaningful differences between patients with vestibular migraine and controls. Although pupillometry remains a promising noninvasive method for exploring autonomic function, its sensitivity and clinical utility for identifying early markers of VM remain uncertain. Further studies involving larger cohorts and complementary neurophysiological approaches are needed to further clarify the role of autonomic modulation and central sensory network dysfunction in VM.

## 6. Limitations

This study has several limitations. First, the sample size was relatively modest, which may have reduced the statistical power to detect subtle differences in pupillary dynamics between the groups. Second, although participants were screened for medications and medical conditions that could affect autonomic function, unmeasured confounding factors, such as hormonal fluctuations or subclinical anxiety, may still have influenced pupillary responses. Third, all measurements were obtained during the interictal phase; therefore, dynamic changes across different phases of migraine were not assessed. Fourth, the cross-sectional design does not allow causal inferences to be drawn regarding the relationship between autonomic dysregulation and VM. In addition, although participants were evaluated during an interictal period defined as at least 72 hours without migraine or vestibular symptoms, it cannot be entirely excluded that some individuals were assessed during the premonitory phase of migraine [29].
